# A Dictionary of Practical Medicine, &c.

**Published:** 1834-01-01

**Authors:** 


A DICTIONARY OF PRACTICAL MEDICINE, &c. By *James Copland*, M.D. Parts 1 and 2, 1832 and 1833. Longman & Co.

THIS meritorious work has reached as far as nearly to the end of D, and has excited a strong sensation throughout the profession, in consequence of the research, industry, and talent displayed by its editor. He has come single-handed to a Herculean labour, where a whole phalanx were already in the field as candidates for the same prize. We long ago predicted that the labours of an individual (if a well-qualified individual could be found), in such an enterprize, would have some peculiar advantages over those of an association of authors—on account of the unity and consistency which these labours would present—and which could not be expected in the rival Cyclopædia, comprising a series of monographs by individuals. Fortunately for the honour and utility of the profession, each work has its peculiar merits and advantages—and the possession of the one ought not, for a moment, to prevent the purchase of the other. The one is a dictionary—the other a cyclopædia. Their plans are different, and their aggregate merits may be pretty equal; but to Dr. Copland, who in his own person matches each individual of the competing association in learning and research, great honour is due, because prodigious energy as well as merit is displayed.

We have found it impossible to give a review of either of these national works, without loading our pages with elementary matter, however valuable in itself, and thus defeating the purpose of a medical journal. Yet we cannot pass over such productions in silence, or afford them merely the niggard tribute of a bibliographical notice. However rigid our adherence to the method of analysis may be, it must bend before the force of such circumstances as these. This is at once our apology and defence of the occasional articles not of an analytical or critical character that appear in the numbers of this Journal. There is seldom a periodical lustrum in which we do not find it necessary to commemorate the publication of some valuable work, too elementary for analysis, too able or too original for neglect. Those of Mr. Lawrence and of Dr. Copland are present and pregnant examples.

The part of the Dictionary of Medicine before us comprises those subjects in medical science which intervene in alphabetical order between "climacteric decay," and "encysted dropsy." Some of the more prominent may be rapidly enumerated:—climate, with its effects on health and on disease—cold, as a remedial or a morbid agent—colic—convulsions—cretinism—croup—debility—delirium—diabetes—the functions and the lesions of the digestive canal—disease, considered in a comprehensive sense—and the several varieties of dropsy.

When we pass from the catalogue of names, to regard more critically and more attentively the execution of the articles, we are struck by the learning displayed by the author. It is worthy of former days, and the patronage of such an undertaking is calculated to diminish the force of the general imputation on the superficial levity of modern writers and readers.

The rich profusion of learning and of literature, and the short space allotted for the present notice, embarrass our choice of an illustrative quotation. Perhaps the following insignificant extract may display the author's manner of handling his subject, as well as more lengthened and more laboured passages, on diseases of a more commanding interest. We select the account of the terminations of diarrhœa, and the morbid appearances seen upon dissection.

"*B.* Diarrhœa may *terminate*—(*a*) in *dysentery*, from an increased affection of the large bowels, frequently connected with inflammatory action or ulceration of their mucous surface and follicles, and spasmodic action of the lower part of the colon: (*b*) or it may run into *enteritis*, or even *peritonitis*, particularly when it commences in the serous form, owing to the extension of inflammation from the internal to the more external coats of the intestines; or to the perforation of them by ulcers; and it may end in abdominal dropsy: (*c*) or it may give rise to *convulsions*, to intus-susceptions, particularly in children: and (*d*) it may assume the *chronic* form, varying in severity and duration, and occasioning mesenteric disease, emaciation, and exhaustion; and it may be prolonged even for years, with irregular remissions and intermissions.

*C.* The *appearances* on dissection can be ascertained only in severe or chronic cases, or in those who have died of its complicated states; or of some other disease on which diarrhœa had supervened, or with which it was associated. In some recent or slight cases, the *mucous coat of the intestines* has been found quite pale and bloodless; and the follicles, only, more developed than usual. In others, it has been somewhat softened, or merely injected; occasionally it has been congested and discoloured, the injection or congestion generally existing in patches or streaks, between which it has been quite pale. In more chronic and severe cases, it has likewise been pale, anœmic, and softened: in some, inflamed, congested, and of every shade, from a rose tint to a brownish or purplish colour —commonly in streaks or patches. In some instances, either without, or in addition to, these and other appearances about to be noticed, the mucous and *submucous* tissues have been œdematous, thickened, and very much softened. Inspissated mucus, oı even coagulable lymph, and more frequently a thin, brownish or greyish, or puriform mucus, have been found covering the diseased surface. In some cases of children, the intestines have become soft, white, almost diaphanous, and easily torn; and have contained a purulent, custard-like matter. Their calibre, in a few instances, has been greater than usual; but much more frequently diminished, or even much and irregularly contracted, particularly in the part chiefly affected. In some instances, small pustules containing purulent matter have been observed, apparently unconnected with the follicles; and, upon breaking, have left merely a slight, superficial, and reddish ulceration, or excoriated-like surface (BRIGHT and myself.) Both the small and large intestines have occasionally presented one or more introsusceptions—sometimes a number, especially in infants and children; and, in fatal cases, soon after weaning, softening, with or without inflammatory appearances, has often also existed in the *stomach* and liver. The intestines have been, in some instances, of a darker hue than natural, externally as well as internally; either in large portions, or throughout, and occasionally in thickly disseminated dots or points. The *mucous glands*, particularly in severe or chronic cases, and those belonging to the mucous and lienteric varieties, have been very generally found either prominent, enlarged, inflamed, or the seat of ulceration, or of a dark or blackish colour, by BRUNNER, STARK, LIEUTAUD, BANG, ABERCROMBIE, BRIGHT, ANDRAL, ANNESLEY, and myself. Fungoid ulcers in the situation of the follicles, often with prominent and inflamed bases, have likewise been observed by these writers. BRUNNER (*De Gland. Duodeni, &c.*) noticed their prominent and enlarged state in the duodenum; and STARK (*Klin. Bemerk, &c.* p. 7.) principally in the large bowels. I have often observed them enlarged, or otherwise diseased, in the former of these situations, in cases of the lientery and atrophy of children; but those of the cæcum, of the termination of the ilium, and of the colon, are more frequently affected in this class of patients. The *mesenteric glands* are often inflamed, or enlarged, or indurated, particularly in young subjects, and in chronic and lienteric cases. The *gall-bladder* sometimes contains greenish bile; and the *liver* is occasionally more vascular than natural. The parts most commonly or most severely diseased are the ilium, especially its lowest third, and the cæcum. The absence of any appreciable lesion in some cases, and the slight nature of those observed in others, militate against the doctrine of BROUSSAIS as to the universal dependence of diarrhœa on inflammation of the intestinal mucous surface. He, however, contends that the blood had retired, in such cases, from the inflamed capillaries into the veins, at the time of, or after, death; thereby leaving no traces of inflammation observable on dissection. This change may occur in vessels that are simply excited, or after erethism merely of the mucous coat (states most frequently attendant upon slight diarrhœa); but not when inflammation has actually existed. (See DIGESTIVE CANAL—*Pathology of.*)"

We have little to add on parting with our author. The success of this Dictionary in not more creditable to him than to the public, and all men of candour and of sense would esteem it a disgrace to the profession and the age, if industry and talent, such as are displayed by Dr. Copland, were passed without attention and without reward. The high estimation of his contemporaries must be gratifying to the feelings of that gentleman, whom we mention with pleasure and with pride as our friend; the significant approbation of the public will form a more solid, if not a more flattering recompense, for his anxious and unremitting toil.

